# Acceptable performance of the Abbott ID NOW among symptomatic individuals with confirmed COVID-19

**DOI:** 10.1099/jmm.0.001372

**Published:** 2021-07-26

**Authors:** William Stokes, Byron M. Berenger, Takshveer Singh, Ifueko Adeghe, Angela Schneider, Danielle Portnoy, Teagan King, Brittney Scott, Kanti Pabbaraju, Sandy Shokoples, Anita A. Wong, Kara Gill, LeeAnn Turnbull, Jia Hu, Graham Tipples

**Affiliations:** ^1^​ Public Health Laboratory, Alberta Precision Laboratories, Alberta, Canada; ^2^​ Department of Pathology and Laboratory Medicine, University of Alberta, Alberta, Canada; ^3^​ Division of Infectious Diseases, Department of Medicine, University of Alberta, Edmonton, Alberta, Canada; ^4^​ Department of Pathology and Laboratory Medicine, University of Calgary, Calgary, Alberta, Canada; ^5^​ Cumming School of Medicine, University of Calgary, Calgary, Alberta, Canada; ^6^​ Faculty of Medicine & Dentistry, University of Alberta, Edmonton, Alberta, Canada; ^7^​ Department of Community Health Sciences, University of Calgary, Calgary, Alberta, Canada; ^8^​ Public Health, Alberta Health Services, Alberta, Canada; ^9^​ Li Ka Shing Institute of Virology, University of Alberta, Edmonton, Alberta, Canada

**Keywords:** COVID-19 diagnostics, ID NOW, rapid SARS-CoV-2 test

## Abstract

**Introduction:**

The ID NOW is FDA approved for the detection of SARS-CoV-2 in symptomatic individuals within the first 7 days of symptom onset for COVID-19 if tested within 1 h of specimen collection.

**Gap statement:**

Clinical data on the performance of the ID NOW are limited, with many studies varying in their study design and/or having small sample size.

**Aim:**

In this study we aimed to determine the clinical performance of the ID NOW compared to conventional RT-PCR testing.

**Methodology:**

Adults with COVID-19 in the community or hospital were recruited into the study. Paired throat swabs were collected, with one throat swab transported immediately in an empty sterile tube to the laboratory for ID NOW testing, and the other transported in universal transport media and tested by an in-house SARS-CoV-2 RT-PCR assay targeting the E gene.

**Results:**

In total, 133 individuals were included in the study; 129 samples were positive on either the ID NOW and/or RT-PCR. Assuming any positive result on either assay represents a true positive, positive per cent agreement (PPA) of the ID NOW compared to RT-PCR with 95 % confidence intervals was 89.1 % (82.0–94.1%) and 91.6 % (85.1–95.9%), respectively. When analysing individuals with symptom duration ≤7 days and who had the ID NOW performed within 1 h (*n*=62), ID NOW PPA increased to 98.2 %.

**Conclusion:**

Results from the ID NOW were reliable, especially when adhering to the manufacturer’s recommendations for testing.

## Introduction

The ID NOW (Abbott) is approved by the United States Food and Drug Administration (FDA) for the detection of severe acute respiratory syndrome-coronavirus-2 (SARS-CoV-2) in individuals who are within the first 7 days of symptom onset. The ID NOW assay uses isothermal nucleic acid amplification of a region of the viral RNA-dependent RNA polymerase (RdRp) to detect the presence of SARS-CoV-2, with results available in under 15 min. Clinical specimens approved for testing include nasal, throat and nasopharyngeal swabs, which must be tested on the Abbott ID NOW either immediately or within 1 h of collection. Specimens placed in viral/universal transport media (UTM) are not valid for testing by the Abbott ID NOW [1].

Current limitations of the Abbott ID NOW include the paucity of strong data to determine its effectiveness in detecting SARS-CoV-2 in clinical settings. The studies used to obtain FDA approval were *in vitro*. These studies demonstrated that the limit of detection of the Abbott ID NOW is similar to other nucleic acid amplification tests at approximately 125 genome equivalents per millilitre. Of the clinical studies reported in the literature, the Abbott ID NOW has been shown to have excellent specificity (~100 %) but sensitivity varies widely between studies (48.0–94.1 %) [2–17]. In addition, many of these studies vary in their study design such as comparing nasopharyngeal to nasal specimens or having major delays in testing specimens on the ID NOW. Some studies were also conducted prior to Abbott’s updated guidance on ID NOW specimen transportation that recommended against using UTM [3]. Only two studies adhered to the FDA’s recommendations for ID NOW specimen collection and transportation, and both studies had a small sample size (<20 samples positive for SARS-CoV-2) [16, 17].

We sought to assess the positive per cent agreement (PPA) of the ID NOW by comparing its performance to an in-house validated real-time reverse transcriptase PCR (RT-PCR) among individuals with recently confirmed COVID-19 while adhering as closely as possible to the manufacturer’s recommendations. We also tested the accuracy of the ID NOW with samples taken from asymptomatic individuals at low risk for COVID-19 (i.e. no exposures), and on retrospective clinical samples previously positive for common respiratory pathogens.

## Methods

### Testing individuals with confirmed COVID-19

Community and hospitalized individuals within the Calgary and Edmonton Health Zones of Alberta, Canada, who recently tested positive for SARS-CoV-2 at Alberta Precision Laboratories (APL) and confirmed as cases by Alberta Health Services (AHS) Public Health were recruited. Diagnostic testing was performed by a Health Canada-approved SARS-CoV-2 assay or a laboratory-developed real-time RT-PCR assay (see below for details). Participants were identified by an AHS Public Health confirmed case list. Oral consent by phone was obtained for collection of samples in the participant’s home or in a hospital (if hospitalized). Individuals under the age of 18 years and individuals in supportive or congregate living facilities were excluded. Individuals who lived farther than a 30 min drive from the laboratory were also excluded. Eligible patients who consented to the study were recruited to have two throat swabs collected by trained healthcare professionals within their homes or inpatient unit.

Individuals were asked to confirm their symptoms and date of symptom onset at the time of study swab collection. Healthcare workers performing the collection were given instructions on how to perform throat swabs using the ClassiqSwabs (COPAN Diagnostics) and the throat swab provided in the ID NOW testing kits (Abbott) [18]. Throat swabs were collected from both sides of the oropharynx and the posterior pharyngeal wall under the uvula. Throat swabs were collected approximately 1 min apart, and the order in which throat swabs were collected was recorded.

For each paired throat swab, one was placed into a dry 15 ml conical centrifuge tube (Fisher Scientific) for ID NOW testing and the other into a tube containing UTM (COPAN) for RT-PCR testing. After testing one household, samples were transported to the APL Public Health Laboratory as quickly as possible, at room temperature, and tested upon receipt. Testing on the ID NOW instrument was done immediately upon receipt as per the manufacturer’s instructions. Throat swabs in UTM collected for RT-PCR testing were stored at 4 °C and tested within 72 h. Two hundred microlitres of UTM was extracted on the MagMAX Express-96 or Kingfisher Flex (ABI) using the MagMAX-96 Viral RNA Isolation Kit (ThermoFisher) or the PurePrep Pathogen Kit (MolGen) according to the manufacturers’ instructions, and eluted into a volume of 110 µl. RT-PCR testing included an assay targeting the envelope (E) gene of SARS-CoV-2, developed and validated at APL, and the Cobas SARS-CoV-2 (Roche Diagnostics) test on the Cobas 6800 instrument (dilution studies compared to other RT-PCR platforms provided in Table S1 within the supplementary material) [19].

For our lab-developed test, the samples were considered positive for SARS-CoV-2 when the cycle threshold (Ct) value was <35. If the Ct was ≥35, amplification from the same eluate was repeated in duplicate and was considered positive if at least 2/3 results had a Ct <41. Testing for SARS-CoV-2 on the Cobas 6800 instrument was performed according to the manufacturer’s instructions. For the Cobas SARS-CoV-2 test, a positive result was defined as 2/2 targets positive or one or more targets positive in duplicate. If 1/2 targets were positive and duplicate testing was negative, the result was considered indeterminate.

For discrepant results, the specimens were retested in triplicate with our lab-developed RT-PCR test and in triplicate with the N2 assay from the CDC 2019-Novel Coronavirus (2019-nCoV) Real-Time RT-PCR Diagnostic Panel using the UltraPlex 1-Step Toughmix (Quantabio) [20]. If still negative, the test was run on the Cobas 6800. PPA was calculated with Clopper–Pearson 95 % confidence intervals. Statistical analysis was performed using a Pearson Chi-squared test for categorical variables and *t*-test for continuous variables using STATA (version 14.1).

### Negative samples and retrospective samples containing other respiratory viruses

Two throat swabs were collected from asymptomatic individuals at low risk of having COVID-19 (no recent travel, no exposures). One throat swab was tested immediately (<2 min) on the ID NOW instrument. The other throat swab was tested by RT-PCR as explained above. To assess for cross-reactivity, retrospective samples containing high concentrations of various respiratory viruses, stored in UTM, were tested by aliquoting 400 µl of sample into the ID NOW blue specimen receiver. These samples were previously tested by either the NxTAG Respiratory Pathogen Panel (Luminex) or the CDC influenza SARS-CoV-2 multiplex assays. The ability of the ID NOW to process this volume of UTM was confirmed by testing four retrospective positive SARS-CoV-2 samples, in UTM, and showing that they could be detected (data not shown, Ct values ranging from 21 to 28).

## Results

In total, 152 patients were recruited for this study. Fourteen individuals were asymptomatic at the time of COVID-19 diagnosis and at time of study collection and were therefore excluded from analysis (sub-analysis provided in the supplementary material). The date of symptom onset for two individuals was not captured at time of consent and they could not be reached later for clarification, and therefore were excluded. Three samples were excluded as samples were lost for RT-PCR testing (all were ID NOW-positive). Symptom details were not recorded for two individuals but they were still included in the analysis as symptom onset was known and they were still symptomatic at the time of collection. This resulted in the inclusion of 133 individuals in our analysis, and their characteristics are provided in Table 1.

**Table 1.** Patient characteristics (*N*=133)

Characteristic	*N* (%)
Male gender	49 (36.8 %)
Mean age in years (median, range)	42.9 (40.4, 19.0–92.7)
Inpatient/hospitalized at time of collection	12 (9.0 %)
ID NOW throat swab collected first	103 (77.4%)
Mean time from sample collection to ID NOW testing (median, range)	54.8 min (54, 20–233 min)
Samples tested on ID NOW within 1 h of collection	83 (62.4 %)
Mean time from starting ID NOW test to confirming positive result (median, range) (*N*=106)	2.4 min (2, 1–8 min)
Mean duration of symptoms from collection date (median, range)	6.9 days (7, 1–17 days)
Individuals with symptom duration ≤7 days from collection date	100 (75.2 %)
Individuals with symptom duration ≤7 days and ID NOW test conducted within 1 h from collection	62 (46.6 %)

All individuals had symptoms at the time of collection and the majority (91.0 %) were from the community. For the 131 individuals with recorded symptoms, cough was the most frequent symptom (40.5 %), followed by pharyngitis (31.3 %), fevers/chills (30.5 %), headache (24.4 %), nasal congestion (23.7 %), anosmia (22.9 %), malaise (21.4 %), myalgias (21.4 %), ageusia (19.1 %), shortness of breath (13.0 %), rhinorrhea (11.5 %), nausea/vomiting (3.8 %), and other including chest pain, diarrhoea, loss of appetite or arthralgias (7.6 %).

Mean duration of symptoms at the time of collection was 6.9 days (median=7, range=1–17 days). Seventy-five per cent (*n*=100) of individuals were within the 7 day symptom onset window, 62.4 % (*n*=83) of individuals had their samples tested on the ID NOW within 1 h of collection, and 46.6 % (*N*=62) met the regulatory agency approved criteria for testing on the ID NOW (symptom onset ≤7 days and ID NOW test conducted within 1 h of collection). The mean E gene Ct value for positive results from the RT-PCR assay was 30.9 (median=31.0, range=16.4–37.9).

Of the 133 samples, 96 were positive on both the ID NOW and the RT-PCR (Table 2). Assuming any positive result represents a true positive, the PPA of the ID NOW compared to RT-PCR with 95 % confidence intervals was 89.1 % (82.0–94.1%) and 91.6 % (85.1–95.9%), respectively. There were 13 false negatives on the ID NOW instrument, with 11/13 (91.7 %) having Ct values >30 on RT-PCR (or indeterminate on the Cobas 6800) and 6/13 (41.7 %) with Ct values >37 on RT-PCR (or indeterminate on the Cobas 6800) (Table 3). Eight of the 13 false negative samples (61.5 %) were from individuals within 7 days of symptom onset, four (30.7 %) were samples that were tested on the ID NOW within 1 h of collection, and only one (7.7 %) was from an individual with symptom duration ≤7 days and had the ID NOW performed within 1 h. Samples tested on the ID NOW instrument were more likely to be positive if the sample was tested within 1 h of collection (*P*=0.031) and from individuals who had lower Ct values on RT-PCR testing (*P*<0.001) (Table 4). RT-PCR samples were more likely to be positive if the sample was tested on individuals with symptom duration ≤7 days (Table S2, available in the online version of this article).

**Table 2.** Results of ID NOW and RT-PCR in symptomatic COVID-19 patients (*N*=133)

		RT-PCR	
		Positive	Negative
**ID NOW**	**Positive**	96	10
	**Negative**	13	14

**Table 3.** Details on the ID NOW-negative, RT-PCR-positive results (*N*=14)

Sample no.	Ct value from RT-PCR	Days from symptom onset at time of collection	Symptoms	Time from sample collection to ID NOW testing (min)
1	33.2	7	Pharyngitis, cough	28
2	37.2	5	Cough, malaise, myalgias, fever	63
3	35.0	5	Pharyngitis, anosmia	79
4	34.3	6	Pharyngitis, cough, malaise, fever	63
5	Indeterminate	6	Pharyngitis, malaise, cough, fever, myalgia, anosmia, ageusia	64
6	31.7	6	Rhinorrhea, fever	94
7	33.8	7	Pharyngitis, cough	66
8	37.0	7	Headache, decreased appetite, anosmia, ageusia	72
9	36.8	8	Malaise	37
10	29.8	9	Sore throat	68
11	37.8	10	Pharyngitis, myalgias	78
12	37.8	11	Shortness of breath	36
13	37.8	12	Cough, anosmia	39
Mean (median)	35.2 (35.9)	7.6 (7)	n/a	60.5 (64)

Only one false negative sample (coloured in grey) was from an individual who had duration of symptoms ≤7 days at time of collection and had their sample tested within 1 h of collection.

**Table 4.** Characteristics between ID NOW-negative and ID NOW-positive samples (*N*=133)

	ID NOW-negative (*N*=27)	ID NOW-positive (*N*=106)	*P*-value
Sample tested within 1 h of collection	44.4 %	67.0 %	0.031
Mean duration of symptoms	7.4 days	6.8 days	0.280
Symptoms ≤7 days at collection	63.0 %	78.3 %	0.099
Mean age	41.4 years	43.3 years	0.590
Mean Ct value	35.2	30.3	<0.001
Throat swab tested on ID NOW collected first	77.8 %	78.1 %	0.972
Hospitalized	11.1 %	8.5 %	0.671
Male gender	44.4 %	34.9	0.359

When tested in triplicate using RT-PCR followed by triplicate testing by the CDC method and testing on the Cobas 6800, 5/10 (50.0 %) RT-PCR-negative samples, with paired positive ID NOW swabs, resolved as positive (Table S3). One sample was unable to undergo additional testing.

**Fig. 1.**
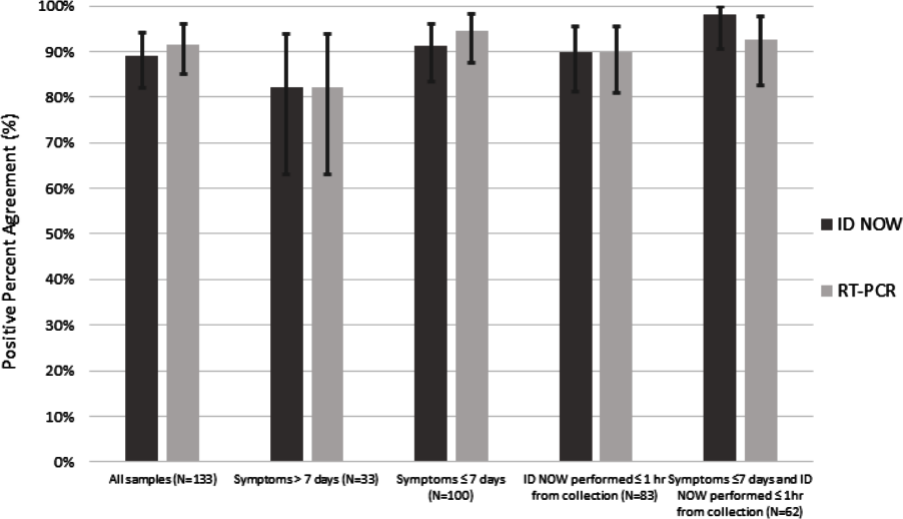
Positive per cent agreement of the ID NOW and RT-PCR from samples, stratified based on sample/individual characteristics, with 95 % confidence intervals provided. Stratified samples include those among all individuals (*N*=133), individuals with symptom duration >7 days (*N*=33), individuals with symptom duration ≤7 days (*N*=100), individuals who had the ID NOW performed within 1 h of collection (*N*=83), and individuals with symptom duration ≤7 days and ID NOW performed within 1 h of collection (*N*=62).

PPA between the ID NOW and RT-PCR, stratified based on individual characteristics, is provided in Fig. 1. The highest ID NOW PPA (98.2 %) was in individuals with symptom duration ≤7 days and who had the ID NOW performed within 1 h, followed by individuals with symptom duration ≤7 days (91.2 %), and in samples with ID NOW performed within 1 h (89.8 %). Further details are provided within the supplementary material (Table S4–S15).

Twenty asymptomatic individuals at low risk of COVID-19 were tested, all of whom were negative on the ID NOW and RT-PCR. All four retrospective samples positive for SARS-CoV-2 were positive on the ID NOW. All 11 retrospective samples containing other respiratory viruses tested were negative. These samples were previously positive for one of either human metapneumovirus, adenovirus, parainfluenza virus, other coronavirus (NL63, HKU1, NL63), enterovirus, respiratory syncytial virus, influenza A H3N2, influenza A H1N1 or influenza B.

## Discussion

Our study compared the PPA of the ID NOW to RT-PCR among individuals confirmed to have SARS-CoV-2 infection. Overall, PPA of the ID NOW instrument was high at 89.1 % and comparable to our laboratory’s RT-PCR (91.6 %) for this population. In the cohort of patients with symptom onset ≤7 days prior to collection, and whose samples could be tested within 1 h of collection, the PPA increased to 98.2 %. This is a result of higher Ct values being observed among those with symptom onset >7 days (Table 3), resulting in decreased PPA of the ID NOW compared to RT-PCR (Table 4). The decreased ID NOW positivity among samples tested after 1 h from collection probably corresponds to viral degradation over time when transported in the absence of viral transport media.

Although our study detected a high PPA of the ID NOW compared to our lab-developed RT-PCR, it is important to note that this is only when testing a population that is actively symptomatic and within the first 7 days of symptom onset. It is not meant to replace standard RT-PCR testing, particularly when testing individuals who are more likely to have lower viral loads, such as individuals with symptom duration greater than 7 days or asymptomatic close contacts. In patient populations where there is a high risk of a severe consequence of missing a case, such as hospitalized or continuing care facilities, RT-PCR or confirming ID NOW negatives with RT-PCR may be warranted. The ID NOW is best suited at the point of care for testing community members who develop early symptoms suggestive of COVID-19, particularly in remote locations where access to standard laboratory testing is limited.

While there were instances in our study where the ID NOW was positive and the RT-PCR was negative, we believe these are true positives for several reasons. Participants recruited in our study were all recently diagnosed with COVID-19; none of the samples from the asymptomatic individuals at low risk of COVID-19 gave false positive results throughout the study; when retested in triplicate or on an alternative platform, 40 % of the discrepant RT-PCR samples had detectable SARS-CoV-2 RNA present; and no issues with false positive results have been identified by the ID NOW manufacturer or among previous publications within the literature [2–17]. We feel that the discrepancies between the ID NOW and RT-PCR are probably related to other factors, such as the variability that comes with collecting multiple specimens.

PPA of the ID NOW varies widely in the literature from 48.0 to 94.1 % [2–17]. However, most of these studies varied in their study design or were conducted when the manufacturer still considered placing swabs in UTM as appropriate for sample collection (prior to April 2020). Several studies did not adhere to the recommended time limit of 1 h for time of collection to result which, as confirmed in this study, is a statistically significant factor in ID NOW’s performance [2, 11, 15]. One of the major studies [3] that detected poorer performance of the ID NOW compared dry nasal swabs (tested on the ID NOW) to nasopharyngeal swabs (tested on Cepheid Xpert Xpress), which is an inappropriate comparison given the superiority of positivity rate among nasopharyngeal specimens to nasal specimens [21]. Furthermore, Basu *et al.* tested patients with symptom onset up to 1 month from time of sample collection, and it is unclear how many patients with confirmed COVID-19 had symptom duration ≤7 days [3]. Among the studies that adhered to current ID NOW manufacturer recommendations for specimen collection and transportation, ID NOW PPA was 66.7 and 94.1 % [16, 17]. However, both studies had small sample sizes and did not provide details as to whether all patients tested were symptomatic and within the first 7 days. The study with ID NOW PPA of 66.7%, for instance, was done primarily for ‘surgical screening’, which suggests that many, if not all, patients were actually asymptomatic.

The limitations of our study include the low number of hospitalized patients recruited such that we cannot make strong conclusions about the ID NOW performance among this population. The majority of throat swabs tested on the ID NOW were collected before the comparator swab (77.4%). However, we did not observe any difference in ID NOW or RT-PCR positivity rate when comparing patients who had ID NOW throat swab collected first vs second (Table 4 and supplementary material). There were discrepancies between ID NOW-positive, RT-PCR-negative specimens that could have resulted from multiple factors, such as intra-collector variability in the throat swab collections and degradation of virus during transportation/storage, as opposed to false positive ID NOW results. Our cross-reactivity study was also limited by using retrospective samples in UTM, as this is not an appropriate transport medium for the ID NOW. We determined that SARS-CoV-2 could be detected by the ID NOW in UTM-containing specimens, but we recognize that some analytical sensitivity may be lost by doing so.

The strengths of our study include the large number of COVID-19-positive individuals recruited, particularly those residing within the community. We adhered to the manufacturer’s recommendations, as much as possible, to recruit COVID-19-positive individuals with symptom duration ≤7 days at time of collection and to test samples on the ID NOW within 1 h of collection. Adhering to these requirements was difficult, as it often took several days from symptom onset for an individual in the community to get swabbed and test results reported. Consequently, symptom onset was often near 7 days. Meeting the 1 h criterion was challenging as we had to drive back and forth from participants’ households to our laboratory, and many participants were located in distant parts of the city (e.g. >30 min drive to the laboratory) and other obstacles (e.g. traffic) increased transit time. Another strength of our study was the concurrent testing of asymptomatic individuals at low risk of COVID-19, and retrospective samples positive for other respiratory viruses, throughout the study to ensure there were no issues with false positive results (e.g. caused by contamination or cross-reactivity).

## Conclusions

The ID NOW was found to be a comparable method to our RT-PCR for the detection of SARS-CoV-2 among individuals with symptomatic COVID-19 infection. PPA was enhanced when tested on individuals with symptom onset ≤7 days and when time from collection to testing was within 1 h. These results reassure us that the ID NOW is a reliable test method in symptomatic individuals, especially when adhering to the FDA-approved indications and recommendations for testing. Given the speed and low complexity of ID NOW testing, these instruments can truly be used as a point-of-care device. As such, they will play an impactful role in combating the COVID-19 pandemic by improving testing in settings where low-volume, rapid (<1 h) turnaround times are much needed, such as among difficult to reach populations (e.g. homeless) and in rural areas where access to a laboratory is limited because transportation delays are significant.

## Supplementary Data

Supplementary material 1
