# Supplementary material for: Acceptable performance of the Abbott ID NOW among symptomatic individuals with confirmed COVID-19
Source: J Med Microbiol. 2021 Jul 26;70(7):001372. doi: 10.1099/jmm.0.001372 (PMC8493423; doi:10.1099/jmm.0.001372)
Supplement: Supplementary material 1 [file jmm-70-1372-s001.pdf]

Supplementary Material: Acceptable Performance of the Abbott ID NOW Among Symptomatic Individuals with Confirmed COVID-19

Table S1: Dilution study comparing limit of detection between various SARS-CoV-2 assays.

	Lab Developed Test	CDC Assay		ID NOW
**quantitated virus**	**E gene**	**N1**	**N2**	**RdRp**
10^{-6}	32.71/33.04/32.56/33.11	31.69/32.31/32.61	31.65/31.87/31.98	Pos
10^{-7}	37.90/36.44/undet/35.7	35.35/36.36/35.39	34.94/34.87/35.11	Pos/Pos/Pos
10^{-8}	37.97/undet/undet/undet	undet/undet/undet	37.82/undet/undet	Neg/Neg/Neg
10^{-9}	undet/undet/undet/undet	undet/undet/undet	undet/undet/undet	-

Assays tested included our lab developed test, US CDC 2019-Novel Coronavirus (2019-nCoV) Real-Time RT-PCR Diagnostic Panel (CDC Assay) and ID NOW (Abbott, Il, United States). Quantitated SARS-CoV-2 virus provided by Public Health Agency of Canada National Microbiology Laboratory.
Undet: undetectable
Neg: negative
Pos: positive

Table S2: Characteristics between RT-PCR negative and RT-PCR positive samples.

	RT-PCR negative (N=24)	RT-PCR positive (N=109)	P-value
**Mean symptoms (days)**	7.8	6.8	0.034
**Symptoms ≤ 7 days**	58.3%	79.2%	0.032
**Mean age (years)**	46.7	42.3	0.236
**Throat swab tested on ID NOW collected first**	79.2	78.1	0.909
**Hospitalized**	16.7%	7.5%	0.163
**Male gender**	45.8%	34.9%	0.316

Table S3: Details on the ID NOW positive, RT-PCR negative results (N=10).

**Number**	**Result when tested in triplicate (lab developed test)**	**Result when tested in triplicate (CDC method)**	**Result when tested on Cobas 6800**	**Duration of symptoms at time of collection (days)**	**Symptoms**
1	U/U/U	U/U/U	U/U	8*	Shortness of breath
2	U/U/U	U/U/U	U/U	12*	Shortness of breath
3	U/U/U	U/U/U	U/U	4	Nasal congestion, anosmia, ageusia
4	U/40.0/U	U/36.5/37.2	33.6/35.7	14	Nausea, malaise
5	U/U/U	35.78/36.87/ 35.82	N/A	4	Nasal congestion, cough, anosmia, ageusia
6	U/U/U	U/U/U	U/U	11	Fever, cough, anosmia, ageusia, pharyngitis
7	U/37.9/U	37.8/38.1/U	N/A	7	Rhinorrhea, cough, anosmia
8	U/36.8/U	36.7/U/U	32.3/34.4	5	Headache, myalgia, malaise
9	U**	N/A	N/A	4	Pharyngitis, headache
10	U/37.9/38.1	U/U/U	N/A	17	Chest pain

Result numbers correspond to E gene Ct value
U = undetectable
*Hospitalized
**Additional testing unable to be performed due to specimen being accidentally discarded

Table S4: Comparison of results from the ID NOW and RT-PCR in COVID-19 patients without symptoms (N=14).

		**RT-PCR**	
		Positive	Negative
**ID NOW**	Positive	7	1
	Negative	1	5

Table S5: Positive percent agreement (PPA) between ID NOW and RT-PCR in asymptomatic COVID-19 patients (N=14). PPA calculated assuming any positive is a true positive.

	**Positive percent agreement**
**ID NOW**	88.9% [95% CI 51.8% - 99.7%]
**RT-PCR**	88.9% [95% CI 51.8% - 99.7%]

CI: confidence interval

Table S6: Comparison of the ID NOW and RT-PCR in hospitalized COVID-19 patients (N=12).

		**RT-PCR**	
		Positive	Negative
**ID NOW**	Positive	7	2
	Negative	1	2

Table S7: Positive percent agreement (PPA) between ID NOW and RT-PCR in hospitalized COVID-19 patients (N=12). PPA calculated assuming any positive is a true positive.

	**Positive percent agreement**
**ID NOW**	90.0% [95% CI 55.5% - 99.8%]
**RT-PCR**	80.0% [95% CI 44.4% - 97.5%]

CI: confidence interval

Table S8: Comparison of the ID NOW and RT-PCR in COVID-19 patients with symptoms > 7 days (N=33).

		**RT-PCR**	
		Positive	Negative
**ID NOW**	Positive	18	5
	Negative	5	5

Table S9: Positive percent agreement (PPA) between ID NOW and RT-PCR in COVID-19 patients with symptoms > 7 days (N=33). PPA calculated assuming any positive is a true positive.

	**Positive Percent Agreement**
**ID NOW**	82.1% [95% CI 63.1% - 93.9%]
**RT-PCR**	82.1% [95% CI 63.1% - 93.9%]

CI: confidence interval

Table S10: Comparison of the ID NOW and RT-PCR in COVID-19 patients with symptoms ≤ 7 days (N=100).

		**RT-PCR**	
		Positive	Negative
**ID NOW**	Positive	78	5
	Negative	8	9

Table S11: Positive percent agreement (PPA) between ID NOW and RT-PCR in COVID-19 patients with symptoms ≤ 7 days (N=100). PPA calculated assuming any positive is a true positive.

	**Positive Percent Agreement**
**ID NOW**	91.2% [95% CI 83.4% - 96.1]
**RT-PCR**	94.5% [95% CI 87.6% - 98.2%]

CI: confidence interval

Table S12: Comparison of the ID NOW and RT-PCR in COVID-19 patients who had the ID NOW tested within 1 hour (N=83).

		**RT-PCR**	
		Positive	Negative
**ID NOW**	Positive	63	8
	Negative	8	4

Table S13: Positive percent agreement (PPA) between ID NOW and RT-PCR in COVID-19 patients who had the ID NOW tested within 1 hour (N=83). PPA calculated assuming any positive is a true positive.

	**Positive percent agreement**
**ID NOW**	89.9% [95% CI 81.0% - 95.5%]
**RT-PCR**	89.9% [95% CI 81.0% - 95.5%]

CI: confidence interval

Table S14: Comparison of the ID NOW and RT-PCR in COVID-19 Individuals with symptoms ≤ 7 days and ID NOW test conducted within 1 hour from collection (N=62).

		**RT-PCR**	
		Positive	Negative
**ID NOW**	Positive	50	4
	Negative	1	7

Table S15: Positive percent agreement (PPA) between ID NOW and RT-PCR in COVID-19 Individuals with symptoms ≤ 7 days and ID NOW test conducted within an hour from collection (N=62). PPA calculated assuming any positive is a true positive.

	**Positive percent agreement**
**ID NOW**	98.2% [95% CI 90.5% – 100%]
**RT-PCR**	92.7% [95% CI 82.7% - 98.0]

CI: confidence interval
